# Current status of *Helicobacter pylori* infection at Fujita Health University Haneda Clinic

**DOI:** 10.20407/fmj.2025-047

**Published:** 2026-05-14

**Authors:** Ryuzo Deguchi, Haruhiko Ogata

**Affiliations:** Department of Gastroenterology, Fujita Health University Haneda Clinic, Ota, Tokyo, Japan

**Keywords:** *Helicobacter pylori* infection, *Helicobacter pylori* eradication confirmation, China

## Abstract

**Objectives:**

In East Asia, the incidence of gastric cancer is high, and *Helicobacter pylori* (*HP*) infection is strongly implicated. China has a high incidence rate of gastric cancer and high prevalence of *HP* infection. We conducted a survey on the current status of *HP* infection in China.

**Methods:**

*HP* antibodies were measured in 496 Chinese nationals who visited our clinic. Participants were classified into groups based on a medical questionnaire: *HP* infection unknown; *HP* non-infected; and *HP* infected. Results of *HP* antibody tests were examined for each group. A comparative analysis was also conducted among 35 Japanese examinees.

**Results:**

The overall *HP* antibody positivity rate in Chinese patients was 39.9% (age group 20s: 14.3%, 30s: 40.0%, 40s: 45.0%, 50s: 35.8%, 60s: 32.6%, 70+: 57.1%). *HP* antibody positivity rates in the *HP* infection unknown, *HP* non-infected, and *HP*-infected groups were 39.0%, 23.3%, and 62.0%, respectively. In the *HP*-treated group, the *HP* antibody positivity rate was 52.3% in the eradication-confirmed group and 69.6% in the non-confirmed group. The overall *HP* antibody positivity rate among Japanese examinees was 17.1%, significantly lower than that among Chinese examinees (*p*=0.0074).

**Conclusion:**

The *HP* antibody positivity rate in Chinese participants was higher among younger age groups, compared with Japanese participants, and tended to be higher when eradication confirmation was not performed after *HP* eradication therapy. Combining the group with unclear *HP* infection status and the group who did not undergo *HP* eradication confirmation, approximately half of Chinese participants had an unclear *HP* infection status.

## Introduction

Gastric cancer is the fifth most common malignant tumor worldwide and ranks as the fifth leading cause of cancer-related deaths.^1^ Five East Asian countries (China, Japan, North Korea, South Korea, and Mongolia) are noted for their high incidence rates of gastric cancer.^2^ Among these countries, China is reported to have the highest incidence, prevalence, and mortality rates. The rate of *Helicobacter pylori* (*HP*) infection in mainland China has shown a declining trend over the past decade but remains high (42.8%) in comparison with Western countries.^3^ *HP* infection is considered the primary cause of gastric cancer, with 90% of patients who have gastric cancer being infected with *HP*.^4^ Eighty percent of patients undergoing health checkups at our clinic are Chinese nationals residing in central to coastal China. In this study, we investigated the current status of *HP* infection and eradication treatment in China using patient questionnaires on gastric cancer and the results from serum *HP* antibody tests. Furthermore, a comparative analysis was conducted among Japanese patients who visited the clinic during the same period.

## Methods

### Participants

A total of 750 patients visited our clinic between October 2023 and May 2025. The present analysis included 496 Chinese nationals (mean age, 50.1 years; 318 men, 178 women) who provided consent for the statistical analysis of their data. As a comparison group, we investigated 35 Japanese individuals (mean age, 58.3 years; 27 men, 8 women) who underwent examination during the same period and provided consent for the statistical analysis of their data. This study was conducted in accordance with the Declaration of Helsinki guidelines.

### *HP* infection history

**Figure 1:**
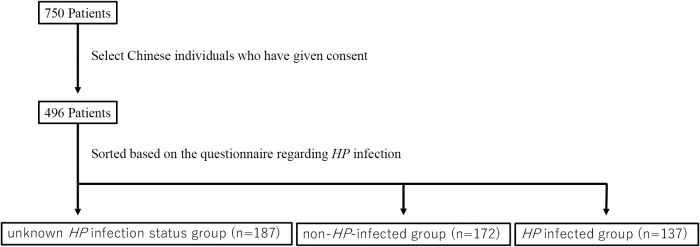
Patient selection flowchart.

Based on questionnaires obtained from study participants prior to their clinic visit, patients were broadly categorized into three groups: a group with unknown *HP* infection status, a non-*HP*-infected group, and an *HP*-infected group (Figure 1). In the *HP*-infected group, patients were classified into those with a history of eradication therapy and those without prior therapy. The group with a history of eradication therapy was further subdivided based on the presence or absence of confirmed *HP* eradication. The non-*HP* infection and *HP* infection groups were classified based on self-reporting. The medical questionnaire was administered by nurses who conducted detailed interviews with each patient regarding *HP* infection (presence or absence of *HP* infection testing and its timing, history of *HP* eradication therapy and its duration, presence or absence of *HP* eradication confirmation, and the testing method used).

### *HP* antibody titer

Serum antibody titers were measured using the latex agglutination turbidimetric method (H. pylori-latex Seiken, Denka Seiken Co., Ltd., Tokyo, Japan). A value of 0–9 U/mL was considered negative and ≥10 U/mL was considered positive.

### Endoscopic diagnosis of *HP* infection

In the confirmation of *HP* eradication, the diagnosis of current infection was made based on the Kyoto classification. Endoscopic findings suggestive of active infection included diffuse redness, mucosal swelling, enlarged fold, sticky mucus, spotty redness, and nodularity. Regarding atrophy, this includes both current and past infections.

## Results

**Figure 2:**
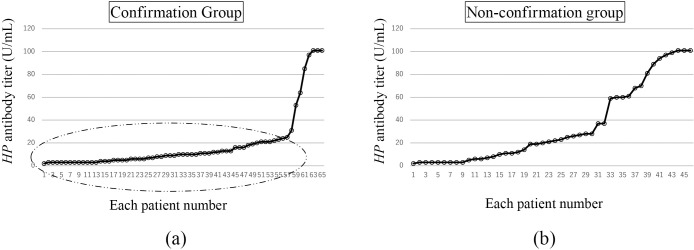
*HP* antibody titer trends based on eradication confirmation status. a: Graph showing antibody titers for the *HP* eradication confirmation group. b: Graph showing antibody titers for the *HP* eradication non-confirmation group.

The overall *HP* antibody positivity rate among Chinese study participants was 39.9% (198/496). *HP* antibody positivity rates by age group were as follows: 20s, 14.3% (1/7); 30s, 40.0% (32/80); 40s, 45.0% (76/169); 50s, 35.8% (44/123); 60s, 32.6% (29/89); and 70s and older, 57.1% (16/28) (Table 1). In the *HP*-infected group, *HP* antibody positivity rates were 73.1% (19/26) in the *HP*-untreated group and 59.5% (66/111) in the *HP*-treated group. The overall *HP* antibody positivity rate among Japanese individuals was 17.1% (6/35), significantly lower than that among Chinese patients (χ^2^ test, *p*=0.0074). *HP* antibody positivity rates among Chinese patients were 39.0% (73/187) in the group with *HP* infection unknown, 23.3% (40/172) in the *HP* non-infected group, and 62.0% (85/137) in the *HP*-infected group (Table 2). The *HP*-infected group displayed a significantly higher positivity rate than the *HP* non-infected group (χ^2^ test, *p*<0.0001). *HP* eradication therapy had been administered to 81.0% (111/137) of the *HP*-infected cohort, and 19.0% (26/137) remained untreated (Table 3). *HP* antibody positivity rates were 59.5% (66/111) in the *HP* eradication therapy group and 73.1% (19/26) in the untreated *HP* group, tending to be higher in the latter group (χ^2^ test, *p*=0.1978). In the group with *HP* eradication treatment, 58.6% (65/111) had undergone confirmation of *HP* eradication and 41.4% (46/111) had not. The age group ≥70 years exhibited the highest rate of eradication confirmation, at 83.3% (5/6), as compared with 0% (0/2) among participants in their 20s (Table 4). The *HP* antibody positivity rate was 69.6% (32/46) in the group without *HP* eradication confirmation and 52.3% (34/65) in the group with *HP* eradication confirmation, with higher positivity rate among patients without confirmed eradication of *HP* (χ^2^ test, *p*=0.0681). Comparing *HP* antibody positivity rates according to the presence or absence of *HP* eradication confirmation across age groups revealed that, except for those aged ≥70 years, participants without *HP* eradication confirmation showed higher positivity rates than those with confirmed *HP* eradication (Table 5). Figure 2 presents a graph in which antibody titers for the *HP* eradication confirmation group and *HP* eradication non-confirmation group are plotted. In the group without *HP* eradication confirmation, a certain increase in antibody titers was observed (Figure 2b). However, in the group with confirmed *HP* eradication, antibody titers remained largely flat up to 30 U/mL (Figure 2a). Patients with antibody titers between 10 and 30 U/mL predominantly represented cases of previous *HP* infection in which eradication was successful. Regarding the results of endoscopy, no findings suggestive of current infection were observed with *HP* antibody titers approximately 20 U/mL; however, we did observe findings suggestive of current infection with titers exceeding 30 U/mL (Table 6). Among the eight patients with *HP* antibody titers 30 U/mL or higher, only one was confirmed to have undergone the urea breath test as the method of confirming *HP* eradication (No. 65); the assessment method for the remaining seven patients was unknown.

The fact that 37.7% (187/496) of study participants had unknown *HP* infection status and 41.4% (46/111) had not undergone *HP* eradication confirmation despite receiving eradication therapy indicates that approximately half of our Chinese participants (47.0%; 233/496) had an unclear *HP* infection status.

## Discussion

Since the International Agency for Research on Cancer classified *HP* as a Group 1 carcinogenic pathogen in 1994, *HP* eradication therapy has been performed worldwide.^5^ The infection rate for *HP* is reported to be 50% worldwide, but this rate varies widely by country.^6^ A meta-analysis reported the annual trends in *HP* infection rates across mainland China from 1983 to 2019, showing a decline from 58.3% to 40.0%,^7^ which closely matched our infection rate of 39.9%.

In Japan, *HP* infection exhibits a birth cohort effect, with infection rates steadily declining as birth cohorts advance.^8^ Reports have indicated a decrease from 59.1% in 1950 to 15.6% in 1990. For the Japanese population born after 1998, the multivariable-adjusted infection rate has been less than 10%. In this analysis of Chinese participants, the *HP* positivity rate exceeding 40% among those in their 30s and 40s suggests that infection is spreading among relatively younger age groups in China, as compared with Japan. *HP* infection rates generally increase with age; however, in our statistical analysis, rates exceeded 40% among those in their 30s and 40s, declining to approximately 30% among those in their 50s and 60s. Reasons for this decline may include the following: 1) the rate of *HP* eradication therapy administration is 68.2% among patients in their 30s but exceeds 80% among those in their 40s to 60s; 2) the rate of *HP* eradication confirmation is 46.7% among individuals in their 30s but ranges from 57.5% to 69.7% among those in their 40s to 50s; 3) the status of oral eradication medication intake is unknown, as patients are not regularly undergoing *HP* eradication confirmation.

*HP* transmission is thought to occur via oral infection, with reports of transmission within families, particularly between mothers and infants or among siblings.^9^ *HP* reinfection is considered rare,^10^ and appropriate eradication therapy can prevent outbreaks. However, antibiotic resistance rates in China are reported to be approximately 20%–40% for clarithromycin and levofloxacin and 60%–90% for metronidazole.^11^ In one review, only 58.6% of patients had undergone *HP* eradication confirmation despite having a history of *HP* eradication therapy. Using the Kyoto Classification^12^ for endoscopic diagnosis of *HP* infection, findings suggestive of active *HP* infection were observed in patients with anti-*HP* antibody titers 30 U/mL or higher; titers in the range of 10–20 U/mL were almost exclusively associated with findings of gastric mucosal atrophy due to past infection. Regarding anti-*HP* antibody titers, values within the range of 3–9.9 U/mL are classified as negative-high titer and have been reported to indicate a high-risk group for gastric cancer development.^13^ In this study, 38.5% (25/65) of cases within the 10–20 U/mL range after infection were considered potential false positives, suggesting that further investigation is needed regarding the reference range for anti-*HP* antibody titers. In the group undergoing *HP* eradication confirmation, even in cases of successful treatment, false positives were present, with antibody titers in the range of 10–30 U/mL. This finding indicates that relying solely on anti-*HP* antibodies for diagnosing active as well as past *HP* infection has limitations, and a clear medical history regarding eradication treatment is considered important. Inadequate administration of *HP* eradication therapy or persistent infection in cases of failed *HP* eradication contributes to the spread of *HP* infection. Therefore, concurrently with prescribing *HP* eradication medication, our hospital schedules follow-up appointments to confirm the eradication of *HP*.

### Limitations

This study focused on patients visiting a single facility and included patients from specific regions within mainland China; therefore, the study findings do not represent the Chinese population as a whole. Furthermore, because *HP* infection was evaluated using serum anti-*HP* antibodies, it was not possible to accurately distinguish between current and past infections.

## Conclusions

Our study population included a group with unknown *HP* infection status comprising 37.7% (187/496) of participants, and 41.4% (46/111) of participants had no confirmation of *HP* eradication, despite receiving *HP* eradication therapy. Consequently, approximately half of Chinese participants (47.0%; 233/496) had unclear *HP* infection status.

## Figures and Tables

**Table 1:** *HP* antibody positivity rate

Age (years)	Chinese	Japanese	*p* value*
20–29	14.3% (1/7)	50% (1/2)	
30–39	40.0% (32/80)	—	
40–49	45.0% (76/169)	0% (0/7)	
50–59	35.8% (44/123)	11.1% (1/9)	
60–69	32.6% (29/89)	11.1% (1/9)	
>69	57.1% (16/28)	37.5% (3/8)	
Total	39.9% (198/496)	17.1% (6/35)	*p*=0.0074

* χ^2^ test

**Table 2:** *HP* antibody positivity rate based on history of *HP* infection

*HP* infection history	*HP* antibody (+)	*HP* antibody (–)
Unknown group (n=187)	39.0% (73/187)	61.0% (114/187)
Negative group (n=172)	23.3% (40/172)	76.7% (132/172)
Positive group (n=137)	62.0% (85/137)	38.0% (52/137)

**Table 3:** Rate of *HP* eradication therapy

Age (years)	*HP* eradication therapy
20–29 (n=2)	100% (2/2)
30–39 (n=22)	68.2% (15/22)
40–49 (n=40)	82.5% (33/40)
50–59 (n=47)	85.1% (40/47)
60–69 (n=17)	88.2% (15/17)
>69 (n=9)	66.7% (6/9)
Total (n=137)	81.0% (111/137)

**Table 4:** Rate of *HP* eradication confirmation

Age (years)	Judgment (+)
20–29 (n=2)	0% (0/2)
30–39 (n=15)	46.7% (7/15)
40–49 (n=33)	69.7% (23/33)
50–59 (n=40)	57.5% (23/40)
60–69 (n=15)	46.7% (7/15)
>69 (n=6)	83.3% (5/6)
Total (n=111)	58.6% (65/111)

**Table 5:** *HP* antibody positivity rate between presence or absence of *HP* eradication confirmation

Age (years)	Confirmation (+)	Confirmation (–)	*p* value*
20–29 (n=2)	0% (0/0)	50.0% (1/2)	1
30–39 (n=15)	57.1% (4/7)	100% (8/8)	0.077
40–49 (n=33)	52.2% (12/23)	70.0% (7/10)	0.025
50–59 (n=40)	47.8% (11/23)	58.8% (10/17)	0.538
60–69 (n=15)	28.6% (2/7)	75.0% (6/8)	0.132
>69 (n=6)	100% (5/5)	0% (0/1)	0.167
Total (n=111)	52.3% (34/65)	69.6% (32/46)	0.08

* Fisher’s exact test

**Table 6:** *HP* antibody titers and endoscopic findings in the *HP* eradication confirmation group

Patient Number	Age	Sex	*HP* antibody titer	Endoscopic findings*
1	32	M	<3	A
2	40	M	3	A
3	41	M	3	None
4	42	M	3	A
5	44	M	3	A
6	44	M	3	A
7	44	M	3	none
8	46	M	3	No endoscopy
9	50	M	3	A
10	50	M	3	A
11	53	M	3	A
12	54	M	3	A
13	62	F	3	A
14	43	M	4	A
15	57	M	4	A
16	63	M	4	A
17	37	M	5	A
18	53	M	5	A
19	55	M	5	A
20	68	F	5	A
21	36	M	6	A
22	44	F	6	A
23	52	M	6	No endoscopy
24	53	F	6	A
25	52	M	7	A
26	54	M	7	A
27	45	M	8	A
28	64	M	8	No endoscopy
29	49	F	9	A
30	52	F	9	A
31	69	M	9	A
32	46	F	10	A
33	49	M	10	A
34	51	M	10	No endoscopy
35	51	F	10	A
36	56	F	10	A
37	35	F	11	A
38	56	M	11	A
39	65	F	11	A
40	46	F	12	A
41	71	M	12	A
42	47	M	13	A
43	72	M	13	A
44	73	M	13	A
45	53	M	16	A
46	59	F	16	A
47	70	F	16	A
48	47	M	18	A
49	39	M	19	A
50	65	F	20	A
51	34	F	21	A
52	44	F	21	A
53	53	F	21	A
54	54	M	22	A
55	46	F	23	A
56	40	M	24	A
57	47	M	25	A
58	42	F	31	M
59	48	F	53	N
60	45	M	64	M
61	74	F	85	S, A
62	53	M	97	S, E, A
63	39	F	101	M, A
64	50	M	101	S, E, A
65	50	M	101	N, S, A

* A; atrophy, M; mucosal swelling, N; nodularity, S; spotty redness, E; enlarged fold
